# Curcumol Inhibits Growth and Induces Apoptosis of Colorectal Cancer LoVo Cell Line via IGF-1R and p38 MAPK Pathway

**DOI:** 10.3390/ijms160819851

**Published:** 2015-08-20

**Authors:** Juan Wang, Fengxiang Huang, Zhun Bai, Bixia Chi, Jiacai Wu, Xu Chen

**Affiliations:** 1 College of Pharmacy, Guilin Medical University, Guilin 541004, China; E-Mails: juanlovelife@163.com (J.W.); huangfengxiang@glmu.edu.cn (F.H.); 2 Research Center for Science, Guilin Medical University, Guilin 541004, China; E-Mails: winatlast@163.com (B.C.); wujiacaiwjc@hotmail.com (J.W.); 3 Intensive Care Unit, Zhuzhou Central Hospital, Zhuzhou 412007, China; E-Mail: baizhun@163.com

**Keywords:** curcumol, colorectal cancer, anti-tumor, IGF-1R, p38 MAPK

## Abstract

Curcumol, isolated from the traditional medical plant *Rhizoma Curcumae*, is the bioactive component of Zedoary oil, whose potential anti-tumor effect has attracted considerable attention in recent years. Though many researchers have reported curcumol and its bioactivity, the potential molecular mechanism for its anti-cancer effect in colorectal cancer LoVo cells still remains unclear. In the present study, we found that curcumol showed growth inhibition and induced apoptosis of LoVo cells in a dose- and time-dependent manner. The occurrence of its proliferation inhibition and apoptosis came with suppression of IGF-1R expression, and then increased the phosphorylation of p38 mitogen activated protein kinase (MAPK), which might result in a cascade response by inhibiting the CREB survival pathway and finally triggered Bax/Bcl-2 and poly(ADP-ribose) polymerase 1 (PARP-1) apoptosis signals. Moreover, curcumol inhibited colorectal cancer in xenograft models of nude mice. Immunohistochemical and Western blot analysis revealed that curcumol could decrease the expression of ki-67, Bcl-2 as well as CREB1, and increase the expression of Bax and the phosphorylation of p38, which were consistent with our *in vitro* study. Overall, our *in vitro* and *in vivo* data confirmed the anti-cancer activity of curcumol, which was related to a significant inhibition of IGF-1R and activation of p38 MAPKs, indicating that curcumol may be a potential anti-tumor agent for colorectal carcinoma therapy.

## 1. Introduction

Colorectal cancer is the third most common cancer and the second cause of cancer death in the Unite States and worldwide [1], and more than 1,200,000 cases were diagnosed and about 600,000 deaths in recent years [2]. It has been recognized as a major public health issue in the Western world. Besides surgical treatment, chemotherapy is the most feasible therapy for colorectal cancer patients. Although the therapy targeting colorectal cancer has improved significantly, it has poor response rates, severe toxicities, high recurrence rates [3], and there is drug resistance to the chemotherapy [4,5]. Therefore, there is an urgent need to search for more effective medicine with fewer side effects for colorectal cancer treatment.

**Figure 1:**
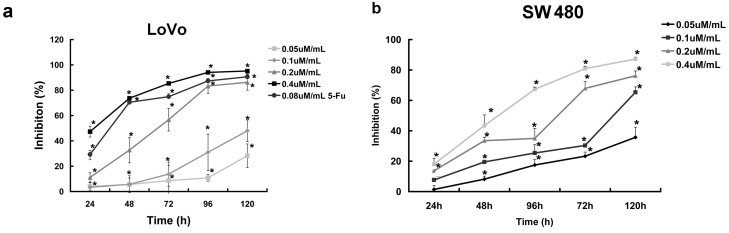
Curcumol inhibited proliferation of human colorectal cancer cell. (**a**) Dose- and time-dependent inhibition effect of cucumol on LoVo cells was evaluated by MTT assay; and (**b**) Dose- and time-dependent inhibition effect of cucumol on SW 480 cells was evaluated by MTT assay. Data represent mean ± SD from at least three independent experiments. * *p* < 0.05 when compared with the untreated control group.

In recent years, more and more cancer therapeutics on the market or in preclinical trails turn to natural products with low toxicity and drug resistance. Chinese people have used the medicinal plant *Rhizoma Curcumae* for thousands of years. Curcumol (Figure 1a) with the structure of a guaiane-type sesquiterpenoid hemiketal, has been reported to possess antitumor, antiproliferation, anti-inflammatory, anti-hepatic fibrosis, antioxidant, and antimicrobial activities with low cytotoxicity [6,7]. Recently, it has been reported that curcumol exhibited growth inhibitory and induced apoptosis activity in several human cancer cell lines *in vitro*, including cervical carcinoma, breast carcinoma, lung carcinoma, gastric carcinoma and hepatocarcinoma [8,9]. Chen found that 300 μM of curcumol could induce HSC-T6 apoptosis, while showing little toxicity to the normal liver cell line BRL-3A [10]. Tang found that curcumol (60 mg/kg) did not cause notable toxicity to the nude mouse [11]. Despite the increasing interest in the anti-tumor activity of curcumol, the mechanisms and signaling path against cancer are still unclear and no studies have investigated how curcumol induces colorectal cancer cell death *in vivo*.

Insulin-like factor-1 receptor (IGF-1R) is involved in many tissues and mainly regulates growth and survival, and is recognized as a unique factor for malignant cells. Thus, IGF-1R blockade can inhibit tumor growth, angiogenesis and enhance chemotherapy-induced apoptosis. Recent studies have demonstrated that IGF-1R plays a crucial role in the oncogenesis and development of colorectal cancer [12,13,14]. Many studies have shown that the activation of IGF-1R is involved in many cellular signaling pathways [15,16], as a tyrosine kinase-containing transmembrane receptor, up-regulation of IGF-IR will relate to several intracellular second messenger pathways, including the mitogen activated protein kinase (MAPK) and phosphatidylinositol 3-kinase (PI3K) pathways [17,18,19]. MAPK signal pathways also play key roles in a number of biological processes [20,21,22]. It is necessary to clarify the regulating mechanism of curcumol on IGF-1R as well as the downstream signal pathway. In this study, we discovered that the anti-tumor effect of curcumol on colorectal cancer *in vitro* and *in vivo*. We found that curcumol inhibited proliferation and induced cell apoptosis in colorectal cancer cells. Moreover, this antitumor activity of curcumol was correlated with the down-regulation of IGF-1R and the up-regulation of p38 MAPK pathways, and finally the activation of PARP-1 cleavage.

## 2. Result

### 2.1. Curcumol Inhibited Cell Proliferation of Colorectal Cancer

To investigate the effect of curcumol on colorectal cancer cells viability, we first examined the inhibition effect of different concentrations of curcumol on the growth of LoVo and SW 480 cells at five different points and then cell viabilities were measured by MTT assay (Figure 1a,b). The result showed that curcumol exhibited a significant inhibition of cell viability in a dose- and time-dependent manner. The 50% growth inhibition of LoVo cells (IC50%) at 24, 48, 72, 96 and 120 h were 0.48, 0.31, 0.24, 0.15 and 0.11 μΜ/mL, respectively. Moreover, we have also detected the long-term effects of curcumol on cell survival using colony-formation assays as described below.

### 2.2. Inhibition of Cell Survival by Colony-Formation Assay

**Figure 2:**
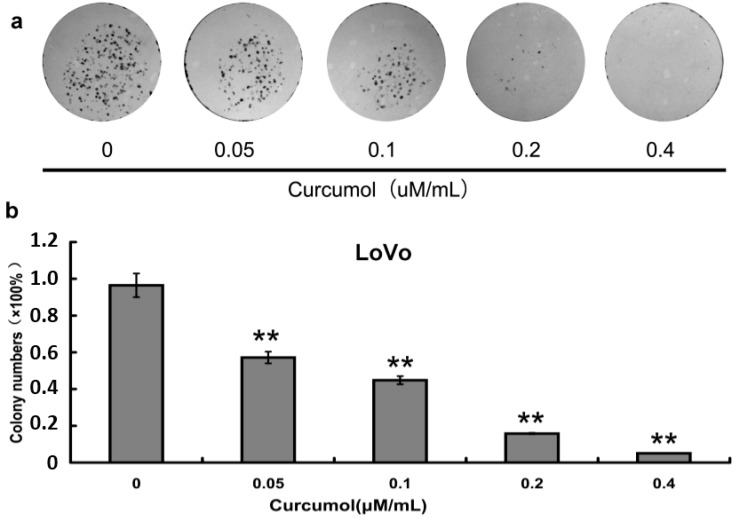
Curcumol inhibited human colorectal cancer cell viability. (**a**) Cell colony formation was evaluated by clonogenic assay; and (**b**) Statistical results of colony-forming assays presented as surviving colonies (percentage of untreated control). Data are expressed as mean ± SD from at least three independent experiments, ** *p* < 0.01 when compared with the untreated control group.

To further evaluate the inhibition effect of curcumol on cell viability, after curcumol treatment colony-formation assay was performed. As shown in Figure 2a,b, curcumol showed a significant inhibition on colony formation in a dose-dependent manner when compared with control group, and at a concentration of 0.4 μΜ/mL curcumol, nearly no colonies were detected. The results from clonogenic assay demonstrated that curcumol could significantly inhibit the colorectal cancer reproductive potentials compared with the control group, which were consistent with the result from MTT assay.

### 2.3. Curcumol Caused Apoptosis in Colorectal Cancer Cells

**Figure 3:**
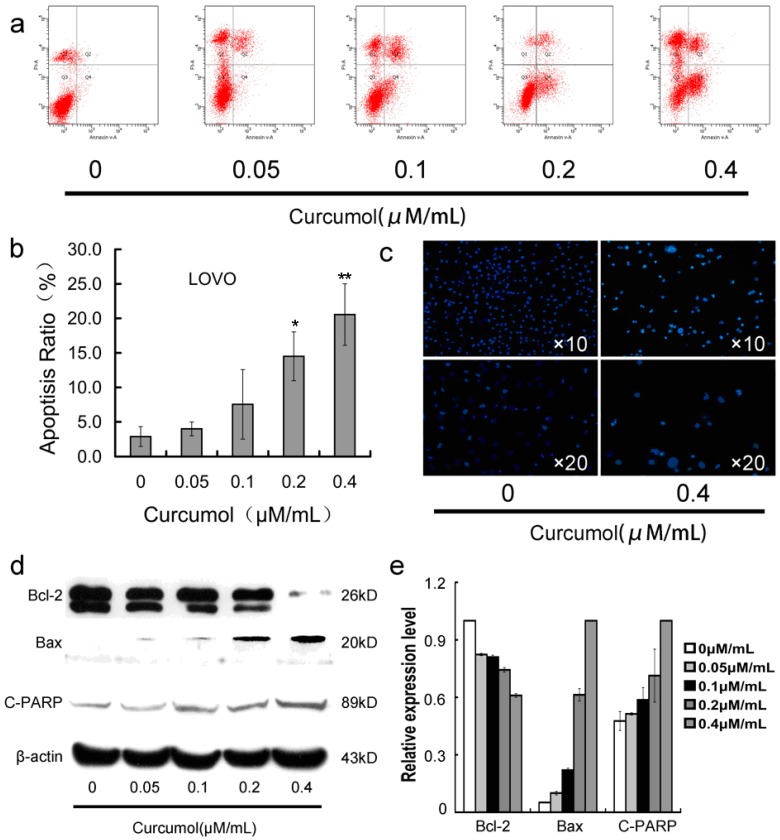
Curcumol induced apoptosis in human colorectal cancer cells. (**a**) Cells were treated with curcumol for 48 h and analyzed by flow cytometry after AnnexinV-FITC/PI staining; (**b**) Statistical results of apoptosis assays by FCM presented as surviving cells (percentage of untreated control); (**c**) Cells were treated with curcumol for 48 h and analyzed after Hoechst 33258 staining; and (**d**,**e**) Bax, Bcl-2 and PARP involved in apoptosis were analyzed by Western blotting. Cells were treated with curcumol for 48 h, and total proteins were extracted. Equal protein loading was evaluated by β-actin. Data are represented as means ± SD from at least of three independent experiments. * *p* < 0.05, ** *p* < 0.01 when compared with the untreated control group.

Furthermore, in order to determine whether the inhibition effect of curcumol on colorectal cancer cells is associated with triggering the programmed cell death pathways, we then analyzed the apoptosis-induction effect of curcumol in LoVo cells. As shown in Figure 3c, the LoVo cells exhibited apoptotic features after treatment with curcumol by Hoechst 33,258 staining. Cells with bright-blue fluorescent condensed nuclei, reduction of cell volume and nuclear fragmentation were obviously observed at 0.4 μΜ/mL curcumol, however, almost none were found in the control group. To confirm the quantity of cell death, Annexin V-FITC and PI fluorescence staining assay were performed by flow cytometry. As shown in Figure 3a,b, curcumol induced LoVo cells apoptosis in a dose-dependent manner at 48 h. Meanwhile, Western blotting results (Figure 3d,e) showed that curcumol decreased the expression of Bcl-2 in an obvious concentration-dependent manner, while significantly increased the expression of bax and cleaved PARP-1.

### 2.4. Curcumol Down-Regulated IGF-1R Levels

**Figure 4:**
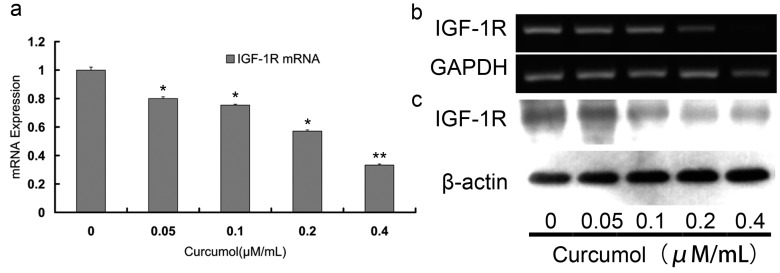
Curcumol down-regulated the expression of IGF-1R in human colorectal cancer cells. The mRNA expression of IGF-1R was detected by real-time PCR (**a**) and RT-PCR (**b**); (**c**) The expression of IGF-1R was analyzed by Western blot. Cells were treated with curcumol for 48 h, and total proteins were extracted. Equal protein loading was evaluated by β-actin. Data are represented as means ± S.D. from at least of three independent experiments. * *p* < 0.05, ** *p* < 0.01 when compared with the untreated control group.

A functional IGF-1R caused anchorage-independent growth in various cancers and activated proliferation and survival signaling pathway [12,23,24]. Numerous studies indicated that IGF-1R may be a promising target for tumor therapy. It has been reported that IGF-1R was overexpressed in more than 90% colorectal carcinomas, which contributed to the malignant characteristics of aggressive growth and poor prognosis [14,25]. To investigate IGF-1R activity in colorectal carcinomas cells following curcumol treatment, the transcription level of IGF-1R was analyzed by the RT-PCR and quantitative real time PCR. We found that increasing concentrations of curcumol decreased IGF-1R mRNA level (Figure 4a,b). Furthermore, we examined the IGF-1R changes at protein level by Western blotting (Figure 4c). As shown in Figure 4c, after treatment with curcumol for 48 h, the protein levels of IGF-1R decreased dose-dependently.

### 2.5. The p38 MAPK Pathways Were Involved in the Anti-Tumor Effects of Curcumol

**Figure 5:**
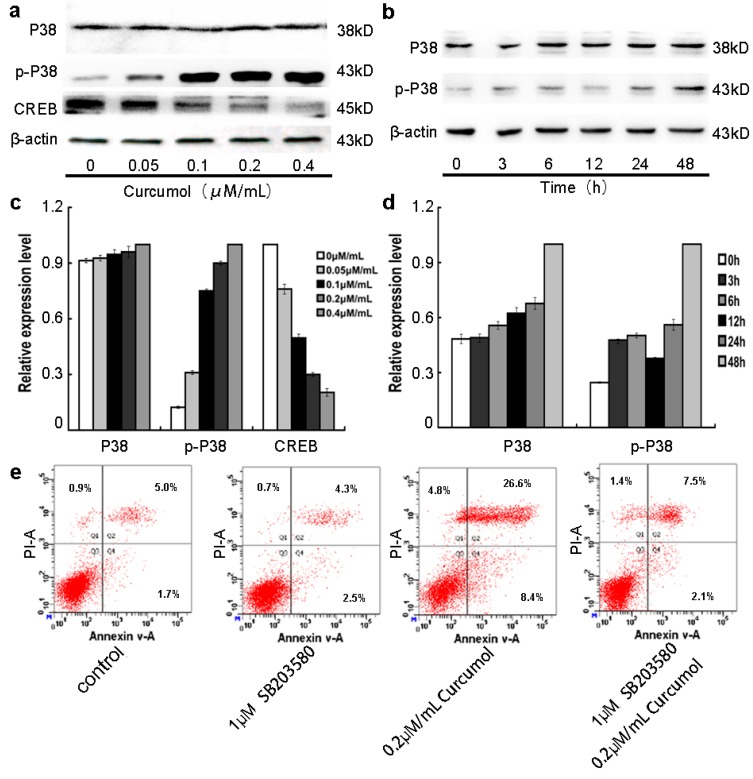
Involvement of p38 MAPK pathway in the anti-tumor effect of curcumol. (**a**,**c**) Cells were treated with curcumol (0, 0.05, 0.1, 0.2 and 0.4 μΜ/mL) for 48 h, and the total proteins were extracted. The expression levels of p38, phospho-p38 and CREB were analyzed by Western blotting; (**b**,**d**) Cells were treated with 0.2 μΜ/mL curcumol for 0, 3, 6, 12, 24, and 48 h. The whole cell lysates were prepared to test the expression levels of p38 and phospho-p38. Equal protein loading was evaluated by β-actin; and (**e**) Cells were treated with SB203580 and curcumol for 48 h after AnnexinV-FITC/PI staining flow cytometry analysis was performed. Representative data are shown from three independent experiments.

MAPK intracellular signaling cascades are involved in the IGF-1R mediated signals pathways [17,24]. It is reported that p38 MAPK acts as a tumor suppressor in various cancer cells [21,26,27], Liu showed that activation of ERK1/2 and suppression of p38 MAPK pathways might be the molecular mechanisms for the malignant behavior of colon cancer cells [28]. Hui has indicated that activation of p38 MAPK could induce apoptosis in colorectal carcinoma cells [29]. To clearly understand the underlying molecular signaling pathways by which curcumol exerted anti-proliferation effect on colorectal cancer cells, we investigated the effects of curcumol on MAPK pathways by Western blot analysis. As shown in Figure 5a,c, we found that increasing concentrations of curcumol significantly increased the expression of phosphorylated p38 and decreased the CREB expression in a dose-dependent manner, while it had no effect on the total levels of p38. Furthermore, we detected the levels of p38 and phosphorylated p38 at the different points. The increase of phosphorylated p38 was observed over time in curcumol-treated LoVo cells (Figure 5b,d). In addition, LoVo cells were treated with p38 inhibitor SB203580. No obvious cell number changes were found in SB203580-treated groups from 0.5 to 5 μM concentration (according the protocol of SB203580). Then, we performed an apoptotic assay by flow cytometry in LoVo cells upon treatment with SB203580, curcumol, or SB203580 combined with curcumol, and the results show that the presence of apoptotic LoVo cells following curcumol treatment (Figure 5e). Combined with the FCM results, our study demonstrated that curcumol induced LoVo cells apoptosis via activation of p38 MAPK and its downstream signal pathway.

### 2.6. Curcumol Suppressed Tumor Growth in Vivo

**Figure 6:**
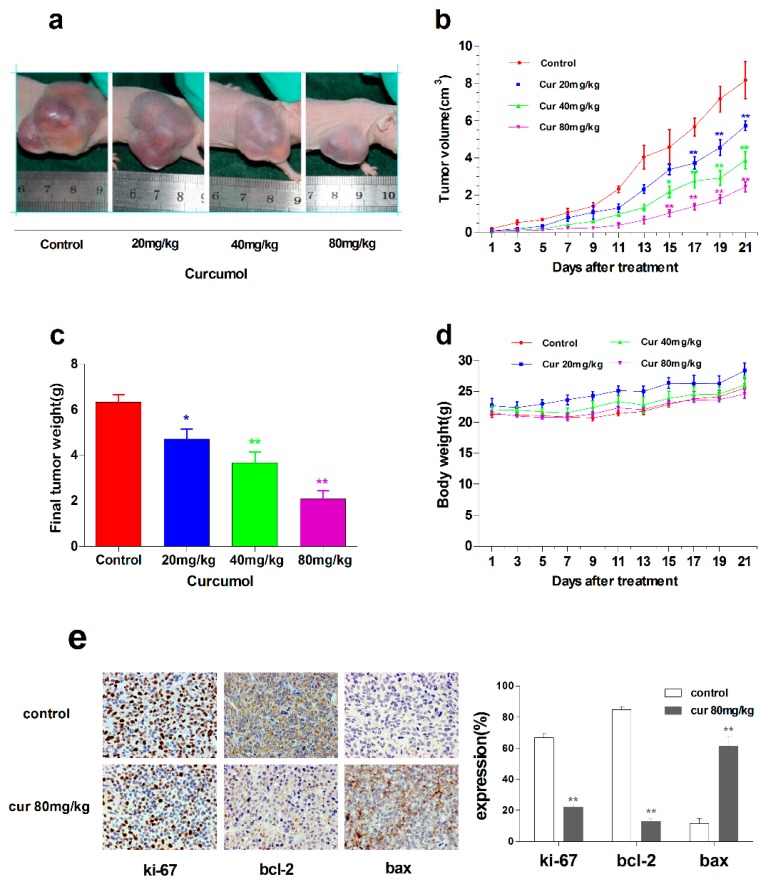

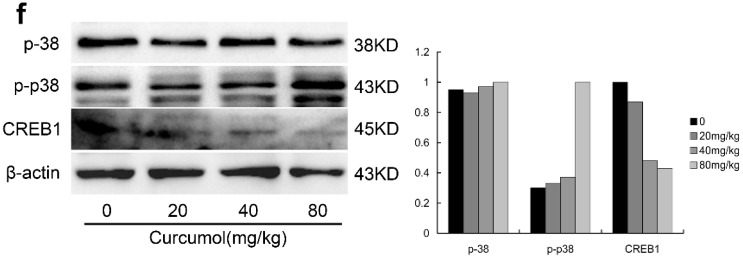
Curcumol suppressed the growth of human colon tumors *in vivo*. Nude mice bearing LoVo human colon xenografts were treated with physiological saline or curcumol at three doses (20, 40, 80 mg/kg of body weight) for 21 days. (**a**) Representative tumor pictures; (**b**) Tumor volume was recorded every three days; (**c**) Tumor weight was evaluated on the twenty-first day; (**d**) Body weight was recorded every three days; (**e**) Immunohistochemical staining of tumor specimens; and (**f**) The level of p38, phospho-p38 and CREB was analyzed by Western blot. Data are represented as mean ± SD; *n* = 6 mice per group. * *p* < 0.05 ** *p* < 0.01 when compared with the untreated control group.

After assessing the anti-tumor activity of curcumol in colorectal cancer *in vitro*, further study was carried out to investigate the anti-tumor effects of curcumol *in vivo*. LoVo cells were xenografted subcutaneously into the right flanks of BALB/c nude mice to establish xenografts. Tumor-bearing mice were treated with vehicle or curcumol at a dosage of 20, 40 or 80 mg/kg/day for the LoVo model. Curcumol substantially suppressed tumor growth in a dose-dependent manner (Figure 6a); significant reductions in tumor volume (Figure 6b) and tumor weight (Figure 6c) were observed in the curcumol-treated groups, and, especially, tumor progression inhibition was nearly 70% in 80 mg/kg group. Furthermore, curcumol-treated groups were well tolerated and did not show significant loss in body weight (Figure 6d).

Consistent with *in vitro* results, ki-67, a proliferation marker of tumors was significantly decreased in curcumol-treated groups. Bax, an apoptosis implication of tumors, was remarkably increased, whereas anti-apoptosis factor Bcl-2 was decreased after curcumol treatment. Furthermore, we found that the expression of CREB1 was inhibited and phosphorylated p38 was up-regulated in curcumol-treated groups (Figure 6f).

## 3. Discussion

A lot of crude medicine has been applied to clinical therapy due to their effectiveness and fewer side effects. Curcumol, a pure monomer, extracted from the traditional Chinese medicine *Rhizoma Curcumae*, has recently been found to have anti-tumor effect on many cancer cells [8,9,30]. Though Zhang’s study showed that curcumol induced apoptosis in lung cancer ASTC-a-1 cells via caspase-independent pathway, but the anticancer mechanism is still unclear. In this study, we evaluated the anti-tumor effect of curcumol on human colorectal cancer cells and investigated the possible underlying mechanism.

Since uncontrolled cell growth is the main biological behavior of malignant tumor [31], we first investigated whether curcumol inhibited proliferation of colorectal cancer cells. Our results showed that curcumol inhibited LoVo cells proliferation in a time- and dose-dependent manner. Apoptosis is a programmed cell death and is a key target in the development of new anti-cancer therapies [32,33]. Curcumol treatment for 48 h could induce LoVo cells apoptosis. The regulation of pro- and anti-apoptotic factor was involved in the progress of cell apoptosis. In the present study, curcumol showed the apoptosis-induction effect with an increase in the Bax/Bcl-2 ratio, and at the same time, curcumol also caused a remarkable cleavage of PARP, which is a marker of early apoptosis.

Numerous evidences have confirmed that IGF-1R was a promising target for cancer therapy [19,34], which was overexpressed in colorectal cancer (CRC), and mediated anti-apoptosis activity in CRC development [24,35]. Due to the important role of IGF-1R in proliferation and survival, inhibition of IGF-1R has been shown to be an efficient approach for cancer therapy [35]. Our results demonstrated that the increasing concentration of curcumol decreased the level of IGF-1R, indicating that IGF-1R was involved in the anti-cancer mechanism of curcumol in LoVo cells.

Valenciano reported that IGF-1R exerted its main action through MAPK and PI3k pathways [19], then blockade of IGF-1R resulted in the changes of the down-stream signals [36], which was involved in p38 MAPK activation [17]. P38 MAPKs have been reported to mediate apoptosis, proliferation and differentiation [1,37,38]. Activation of p38 MAPK in several tumor cells induces apoptosis depending on the cellular context, cancer stage and the stimuli [39]. Gupta found that p38 MAPK deficiency resulted in tumorigenesis in colon cancer [39]. Many studies suggested that p38 MAPK was a negative regulator of malignant transformation and showed pro-apoptotic effects in tumor cells [22,40,41]. Inhibition of p38 MAPK could enhance tumor development *in vivo* or induce resistance to chemotherapy [4,38,42]. However, upregulation of p38 MAPK induced cell cycle arrest or apoptosis [43]. Diane demonstrated that PBA could suppress human lung carcinoma cell growth via activation of p38 [44]. In A549, U2OS, BXPC3 and PANC-1 Cell lines, oleanolic acid exerted certain anti-tumor activity by inducing apoptosis which required the p38 activation [45,46]. In human colonic carcinoma, many studies have revealed that activation of p38 by the berberine or garlic-derived compound s-allylmercaptocysteine could cause tumor cells apoptosis [40,47]. However, it is still unknown whether p38 MAPK pathway is involved in the anti-tumor activity of curcumol on CRC cells. Here, we found that the phosphorylated p38 MAPK was enhanced by up-regulation of phosphorylated p38 MAPK in curcumol-treated CRC LoVo cells. However, inactivation of p38 caused the decrease of the pro-apoptosis effect upon curcumol treatment. Moreover, p38 MAPK exerted pro-apoptosis functions by direct or indirect regulating apoptosis-related factors, such as CREB, Bcl-2, Bax, PARP and so on [5,29,35,48,49]. Curcumol treatment resulted in an increased expression of Bax, a decreased expression of CREB1 and Bcl-2 in LoVo cells. Meanwhile, we also found the cleavage of PARP in a dose-dependent manner, which was consistent with our previous study in lung cancer [50]. As we all know, the Bcl-2 family is engaged in the apoptosis progress in nearly all cancer cells. Either inhibiting the expression of Bcl-2 or enhancing the expression of Bax could be an effective approach for cancer therapy. Our previous work indicated that curcumol induced tumor cells apoptosis via down-regulation of Bcl-2, while the mechanism is not very clear [30]. Hui showed that CREB, which is a direct substrate of p38 MAPK, regulated Bcl-2 expression [29]. In our current study, we also found that curcumol inhibited the expression of Bcl-2 as well as CREB, while it is not been verified whether CREB directly regulated expression of Bcl-2, which was involved in the curcumol-induced apoptosis, and then stimulate the downstream apoptosis signal. Thus, more work needed to be performed to find the defined mechanism of curcumol-induced apoptosis, which is a main focus of our further study.

To investigate the effect of curcumol *in vivo* and find an appropriate dose in cancer therapy, we established colorectal cancer subcutaneous tumor model in nude mice. We treated the mice with three different doses (20, 40 and 80 mg/kg) by intraperitoneal injection every day. We discovered that the tumor volume was markedly attenuated in curcumol-treatment groups, and no adverse effects were observed on body weight and activity compared with the control group. There was no any pathological change in the kidney and liver tissue of curcumol-treated mice compared with the control group by hematoxylin-eosin (HE) staining. Moreover, the level of Bax and phosphorylated p38 were increased by immunohistochemical and Western blot analyses, while the level of Bcl-2 and CREB1 were decreased in curcumol-treated groups, which were consistent with *in vitro* apoptosis effect of curcumol.

In conclusion, these signaling cascades ultimately contributed to the curcumol-induced apoptosis in LoVo cells. In this paper, we investigated the anti-proliferative effects of curcumol on colorectal cancer *in vitro* and *in vivo*, revealing the potential molecular mechanism of curcumol in CRC LoVo cells. The main findings also included the following: (1) Our work presented evidence to demonstrate that curcumol regulated the expression of IGF-1R in colon cancer cells; (2) Curcumol induced LoVo cell apoptosis via an IGF-1R/p38 MAPK signal pathway; (3) Curcumol could suppress the growth of human colorectal cancer xenografts *in vivo*. All of the above findings suggested that curcumol might be used as a potential anti-cancer agent in colon cancer treatment.

## 4. Experimental Section

### 4.1. Ethics Statement

This study was carried out in strict accordance with the institutional guidelines of Guilin medical college (GLMC) Animal Care and Use Committee. The protocol was approved by the Committee on the Ethics of Animal Experiments of GLMC (Permit Number: 35).

### 4.2. Drugs and Reagents

Curcumol (lot: 100185-200506) was obtained from National Institute for the Control of Pharmaceutical and Biological Products (Beijing, China). 3-(4,5)-dimethylthiahiazo(-z-y1)-2,5-*di*-phenytetrazolium bromide (MTT) and dimethyl sulfoxide (DMSO) were purchased from Amerso Chemical Co. (Amresco, Cochran Solon, OH, USA). Propidium iodide (PI) and Annexin V-FITC apoptosis detection kit were purchased from BD Biosciences **(**Bedford, MA, USA). Hoechst 33258 was purchased from Kaiji Biotechnology (Nanjing, China). The antibodies against Bax, PPAR and p-p38 were obtained from Wanlei Biotechnology Company (Shenyang, China), IGF-1R and p38 were purchased from Abcam Company (Cambridge, UK). Antibody β-actin and Bcl-2 were purchased from Santa Cruz Biotechnology Company (Santa Cruz, CA, USA).

### 4.3. Cell Culture

Human LoVo and SW 480 cell lines were obtained from the Guilin medical university (Guilin, China). Cells were cultured in DMEM medium (Gibco BRL, Grand Island, NY, USA) supplemented with 10% fetal bovine serum (FBS; Gibco, Auckland, New Zealand), 100 units/mL penicillin and 100 units/mL streptomycin under humidified conditions with 5% CO_2_ at 37 °C. Cells without curcumol treatment served as a control group. Curcumol was dissolved in the absolute ethyl alcohol as a 10 mg/mL, and then diluted by the 10% FBS DMEM medium.

### 4.4. Cell Proliferation Assay

The sensitivity of cells to curcumol was measured using the MTT assay. Briefly, 800 cells/well were plated in a 96 well plate. The next day, cells were treated with different concentrations of curcumol (0.05, 0.1, 0.2 and 0.4 μΜ/mL), a positive control (0.08 μΜ/mL 5-fu) and negative control (1% of anhydrous alcohol culture medium). After 24, 48, 72, 96 and 120 h, 20 μL/well of 5 mg/mL MTT solution was added to each well and further incubated for 4 h at 37 °C. The formazan crystals formed in the wells were solubilized by adding solubilization solution and incubating the plates at 37 °C 30 min with slight shake. The plates were read at 490 nm on a TECH M200 plate reader (TECH, Switzerland). The percentage cell growth inhibition for each treatment group was calculated by adjusting the untreated control group to 100%. All experiments were repeated at least 3 times.

### 4.5. Colony Formation Assay

To test the survival of LoVo treated with curcumol, the cells were plated 4 × 10^4^–8 × 10^4^ per well) in a six-well plate and incubated overnight at 37 °C. After 48 h exposure to various concentrations of curcumol, then the cells were trypsinized, and the viable cells were counted and plated in 100 mm Petri dishes in a range of 100 to 1000 cells to determine the plating efficiency as well as assess the effects of treatment on clonogenic survival. The cells were then incubated for 10 to 12 days at 37 °C with 5% CO_2_. The colonies were fixed by 4% paraformaldehyde solution, stained with Giemsa stain and then counted. The surviving fraction was normalized to untreated control cells with respect to clonogenic efficiency.

### 4.6. Morphological Analysis after Hoechst Staining

Morphological changes associated with apoptosis in LoVo cells were detected by Hoechst 33258 staining. Briefly, the cells were plated in 6-well plates for 12 h and then treated with various concentrations of curcumol for another 48 h. After treatment, cells were washed with cold PBS and fixed in paraformaldehyde solution for 20 min. The cells were stained with Hoechst 33258 solution (5 μg/mL) followed by PBS washing and examination under fluorescence microscope (olympusIX71FL, Tokyo, Japan) to identify the nuclear morphology of apoptotic cells.

### 4.7. Apoptosis Analysis by Flow Cytometry (FCM)

To further confirm that curcumol could induce cell apoptosis by flow cytometry using Annexin V-FITC apoptosis detection kit. To put it simply, after treatment with different concentrations of curcumol for 48 h as described above, cells were harvested and washed with cold PBS twice. After centrifugation, cells were stained with Annexin V-FITC and PI, and then analyzed with FCM (Becton Dickinson, San Jose, CA, USA).

### 4.8. RT-PCR and Real-Time PCR Analysis

Total RNA was extracted from LoVo cells with Trizol reagent (TIANGEN, China), followed by being reversely transcribed into cDNA via M-MLV first strand RT Kit (Invitrogen, Carlsbad, CA, USA) according to the manufacturer’s instructions. QPCR was performed using SYBR premix Ex Taq (ABI) on 7500 fast Real-Time PCR Detection System (ABI, Foster, CA, USA) supplied with analytical software. The following primers were used: upper IGF-1R 5′-CCATTCTCATGCCTTGGTCT-3′; lower IGF-1R 5′-TGCAAGTTCTGGTTGTCGAG-3′ [23]; upper GAPDH 5′-ACCACAGTCCATGCCATCAC-3′; lower GAPDH 5′-TCCACCACCCTGTTGCTGTA-3′ [15].

### 4.9. Western Blot Analysis

After treatment with the different concentrations of curcumol for 48 h, cells were lysed in RIPA lysis buffer (Beyotime, Shanghai, China) with protease and phosphatase inhibitor cocktail tablets (Roche, Mannheim, Germany). Then, lysates were centrifuged at 12,000× *g* for 25 min at 4 °C. The supernatant was harvested and the protein concentration was measured by the BCA protein assay kit (Beyotime). An equivalent amount of protein was resolved by sodium dodecyl sulfate-polyacrylamide gel electrophoresis (SDS-PAGE) and transferred to a nitrocellulose membrane. Membranes were blocked for 1 h in 5% (*w*/*v*) skim milk in PBST (with 0.1% Tween 20) at room temperature, and then incubated at 4 °C overnight with primary antibodies in PBST with 5% skim milk. Horseradish peroxidase-labeled anti-mouse or anti-rabbit secondary antibodies were added for 1 h at 37 °C and detected with enhanced chemiluminescence (ECL) reagent (BIO-RAD, Hercules, CA, USA), and exposed to medical X-ray film. The optical density of protein bands was measured using Image J software (BIO-RAD). The optical density of each band was normalized by β-actin optical density.

### 4.10. Xenograft Tumor Models

Five-week-old BALB/c athymic nude mice weighting approximately 20 g were purchased from Shanghai SLAC Laboratory Animal Co., Ltd. (Shanghai, China) and were caged in standard laboratory conditions. LoVo tumor cells resuspended in 200 μL PBS and were injected subcutaneously in the right flanks of nude mice. When tumors reached an average volume of 100 mm^3^, the mice were randomly assigned to four groups. Curcumol was dissolved in 90% propylene glycol. Three groups of nude mice were treated with 200 μL curcumol in 90% propylene glycol by intraperitoneal injection at a series dose of 20, 40, 80 mg/kg per day, respectively, and 200 μL 90% propylene glycol were injected into the last group and used as the control. Tumor size and body weight were measured by periodic measurements with calipers every three days, and clinical symptoms were observed daily. Tumor volume was calculated using the following formulae: V = (length × width^2^/2) [3]. After 3 weeks treatment, mice were killed and tumor tissues were embedded in paraffin for further analysis. All surgery was performed under sodium pentobarbital anesthesia, and all efforts were made to minimize suffering.

### 4.11. Statistical Analysis

All data are presented as mean ± SD of at least three independent experiments. ANOVA, followed by multiple comparison *post hoc* test, or Kruskal–Wallis, was used to assess the statistical significance in difference groups. *p* < 0.05 indicated a statistically significant difference.

## Abbreviations

CRC: colorectal cancer
IGF-1R: insulin-like factor-1 receptor
CREB: cAMP-response element binding protein
PARP-1: Poly (ADPribose) polymerase 1
MAPK: mitogen activated protein kinase
P13k: phosphatidylinositol 3-kinase.
